# Patient-Reported Outcomes and Satisfaction after Cervical Epidural Steroid Injection for Cervical Radiculopathy

**DOI:** 10.31661/gmj.v8i0.1478

**Published:** 2019-11-03

**Authors:** Masoud Hashemi, Payman Dadkhah, Mehrdad Taheri, Mahshid Ghasemi, Ali Hosseinpour, Mojtaba Farjam

**Affiliations:** ^1^Anesthesiology Research Center, Shahid Beheshti University of Medical Sciences, Tehran, Iran; ^2^Taleghani Hospital, Shahid Beheshti University of Medical Sciences, Tehran, Iran; ^3^Non-Communicable Disease Research Center, Fasa University of Medical Sciences, Fasa, Fars, Iran

**Keywords:** Radiculopathy, Neck Pain, Steroids, Injection, Epidural, Pain Management

## Abstract

**Background:**

Cervical radiculopathy caused by disc herniation is a frequent public health issue with economical and socio-professional impacts. The objective of the present study is to evaluate the patient-reported outcomes and satisfaction from cervical epidural steroid injection during a 2-year follow-up.

**Materials and Methods:**

Results based on patients’ reports from a previously performed intervention of cervical epidural steroid injection on patients with cervical radiculopathy due to cervical disc herniation are prospectively collected. Outcome measures are Neck Disability Index (NDI), numerical rating scale (NRS) for pain assessment, and 5-scale patient satisfaction questionnaire (PSQ) plus opioid medication for pain relief, additional injections, and progression to surgery.

**Results:**

Of total 37 cases, 34 were available for follow-up after 2-year postoperatively. The mean preoperative NDI was 21.17 and improved to 17.38, and the mean NRS was 7.7 and improved to 5.00; both were statistically significant. Mean patient satisfaction after 2 years was 3.17 out of 5. 11 cases needed additional injections, and 4 of patients proceeded to surgery.

**Conclusion:**

We showed that transforaminal cervical epidural steroid injection for cervical radiculopathy is an effective non-surgical treatment option, providing significant pain relief and functional improvement during 2-years follow-up along with higher-than-average patient satisfaction in most of our patients.

## Introduction

Cervical radiculopathy caused by disc herniation is a frequent public health issue with economic and socio-professional impacts. A combination of multiple pathological processes including disc degeneration, disc material herniation along with the formation of osteophytes may cause neck pain and radiculopathy by mechanical compression or irritation of the nerve root in the foraminal canal and the vertebral artery passing through the intervertebral and transverse foramina [1, 2]. Compression of the nerve root may be caused by disc herniation with or without extruded disc fragments, and degenerative cervical spondylosis [3]. Depending on the disc level, nerve root and disc space involved, clinical presentation may include pain, sensory or motor deficits, diminished reflexes, or a combination of the above [4, 5]{Wainner, 2000 #116;Hashemi, 2018 #127}. Cervical radiculopathy caused by disc herniation is generally a self-limiting condition, and its natural course is generally favorable and in the absence of myelopathy or significant muscle weakness all patients should be treated conservatively for at least six weeks [5]. Conservative treatments consist of neck immobilization, behavior modification, anti-inflammatory medications, physical therapy, and cervical traction are used to enlarge the neural foramen and reduce physiologic neck stress [2, 6, 7]. But after the failure of conservative treatment for compressive cervical radiculopathy and progressive or profound motor weakness, interventional management either surgical or nonsurgical such as epidural injections are considered in pain management of cervical radiculopathy [8-10]. Lumbar epidural steroid injection is now an established and commonly used treatment modality for the management of chronic low back pain and sciatica due to lumbar disc protrusion, and cervical epidural steroid injection was derived from this technique and has been applied as a treatment option for cervical radiculopathy [11, 12]. However, far too little attention has been paid to this new modality, and the paucity of evidence exist in terms of prospective studies regarding long-term patients’ outcome after receiving cervical epidural steroid injections in the management of neck pain and functional ability score improvement [13]. Therefore, the present study was designed to evaluate the effectiveness of cervical epidural steroid injections based on patients’ reports with a 2-year follow-up who had previously undergone cervical epidural steroid injection due to cervical radiculopathy secondary to disc herniation.

## Materials and Methods

The present prospective study was designed and conducted in an interventional pain management referral center (Tehran, Iran), during 2018 to evaluate results based on patients’ reports from a previously performed intervention of cervical epidural steroid injection on patients with cervical radiculopathy due to cervical disc herniation. The study protocol was approved by the local ethics committee of the university (ID: IRSBMURETCH1397833), and the study was performed in accordance with the ethical standards of the 1964 Declaration of Helsinki. The study population was all patients who had undergone cervical epidural steroid injection in our medical institution during 2016.

### Inclusion and Exclusion criteria

Inclusion criteria were age>18 years, history of neck pain and radiculopathy for more than six months due to cervical intervertebral disc herniation which was confirmed by diagnostic medical imaging, and no response to conservative treatments such as physical therapy, reduction in activities and anti-inflammatory medications in last six months. Exclusion criteria were the history of previous spinal surgery, cervical myelopathy, cervical ossification of the posterior longitudinal ligament, kyphotic deformity or diminished vertebral canal diameter, history of psychological illness, alcohol or drug abuse and inability to communicate in the Persian language. Sampling was done using census method, and all the participants of the previous study who met the criteria were included in the present study. Baseline and demographic characteristics for participants were recorded in each patient’s profile, and each participant was called by an independent researcher. If any patient was unavailable after three calls in different times of a day and different days of a week, the patient was excluded from the study. During the phone call interview, Study aim and objective were described to each patient and participants were instructed to respond to the questions or rate each scale independently. Outcome measures were classified as pain intensity and neck disability index, assessed at baseline and 24 months following the treatment. Patients’ satisfaction from the intervention and current opioid intake were also evaluated. Additional cervical spine injection and progression to surgery during the past two years were secondary outcome measures.

### Pain Intensity

The pain intensity was evaluated based on the verbal numerical rating scale (NRS). NRS is one of the most commonly used self-report scales for measuring pain, likely due to its ease of use (it requires no specialized equipment) and because its 0 to 10 metric is preferred by health care professionals. Patients typically were asked, “How strong is your pain during the past 14 days, where 0 is no pain, and 10 is the strongest or worst pain you can imagine?”. The validity and reliability of this scale have been previously established [14].

### Functional Ability

Functional ability was evaluated based on the Neck Disability Index (NDI). The NDI is a 10-item self-administered disease-specific questionnaire evaluating the effect of neck pain on a patient’s daily life and the corresponding disability. The questionnaire ranges from 0 to 50; the higher the score, the greater the disability. The validity and reliability of this scale have been previously established [15].

### Patient Satisfaction

Patient satisfaction was registered at 2-year follow-up and categorized as excellent to poor based on patients’ satisfaction questionnaire (PSQ) among five choices (totally satisfied, partially satisfied, uncertain, partially dissatisfied or totally dissatisfied). The validity and reliability of this scale have been previously established [16]. Patients were asked about their opioid consumption for their presenting symptoms during the past two weeks. Additional cervical spine injection and progression to surgery during the past two years were asked, and the answers were documented in their profile. Clinical outcomes were obtained by an independent and blinded interventionist member of the research team. Due to the long duration of follow-up and for reasons such as death, migration, or change in the status of sample cases over time, the presence of cases with no follow-up (loss-to-follow-up) is predictable. To minimize this bias, inclusion, and exclusion criteria are limited, and therefore, the samples will be completely homogeneous from the pathological point of view. As a result, the sample population will represent the community studied. In order to avoid recall bias, the primary outcome measured concentrated on the current condition of the patients (specifically past two weeks). In order to avoid response bias, patients were provided with adequate details and necessary clarifications about the questions and the correct way of responding to the questionnaires.

### Data Analysis

Data were analyzed using SPSS (version 18, SPSS Inc., Chicago, IL, USA). Continuous variables are presented as mean ± SD and median (range) according to the normal or not-normal distribution of data. Ordinal data are presented as count (%). Paired t-tests were used to compare the variables at baseline and 2-year follow-up between patients. P-value<0.05 was considered to be statistically significant.

## Results

During the past recruitment period, a total of 37 subjects with cervical radiculopathy had undergone cervical epidural steroid injection in our tertiary medical center. After two years of follow-up post-procedure, 34 (91.8%) of the 37 subjects were available. Baseline and demographic characteristics of the participants were analyzed. Mean age of participants was 52.32 years, ranged between 28-75 years. Twelve subjects were male (32.4 %), and 20 cases (54.1%) reporting no comorbidity. Of 17 cases who had reported comorbidity, 2 cases were known cases of hypertension (HTN), 4 were known cases of diabetes mellitus (DM), 2 were known case of ischemic heart disease (IHD) and 5 cases were previously diagnosed with hypothyroidism; while 1 patient reported was diagnosed with DM and HTN, 1 reported was diagnosed with IHD and HTN, 1 reported was diagnosed with IHD and DM, and 1 patient was diagnosed with DM, HTN, and IHD at the same time. C5-6, C6-7 was the most common level of herniation. Baseline data are demonstrated in Table-1. At 2-year follow-up, there was a statistically significant reduction in NDI from 21.17 points preoperatively to 17.38 points postoperatively (P=0.021). Neck pain measured by NRS was reduced from 7.76 preoperatively, to 5 postoperatively, a reduction of 2.76 which was statistically significant (P=0.04). When analyzed for patient satisfaction we found that 16.2% of the patients were totally satisfied, 18.9% were uncertain, and 18.9% were totally dissatisfied with the intervention outcome at 2-year follow-up. Mean patient satisfaction score (PSQ) was 3.17 while 17 cases (50 %) reported a PSQ level ≥4. Of those who reported having current pain (37 cases), 7 cases (20.6%) reported using opioid for analgesia, 11 cases (32.4%) reported receiving additional injections, and 4 cases (11.8%) reported having undergone surgery (Table-2). Of the 30 cases (91.8%) who did not undergo surgery, all of them reported current pain, 4 (13%) reported current opioid medication usage and 8 (26%) reported receiving additional cervical injections. Of 4 cases (8.2%) who reported receiving surgery, all of them reported current pain. Additionally, 3 reported using current pain medications (75%), and 3 (75%) underwent additional injections. Comparison of outcomes stratified by the pursuit of surgery are demonstrated in Table-3.

## Discussion

In our study, the patient-reported outcomes after 2-year follow-up showed statistically significant improvement in NRS and NDI postoperatively with an average patient satisfaction rating (PSQ: 3.17/5). Cervical epidural steroid injection has demonstrated clinical success with high patient satisfaction in earlier studies [13, 17-20] but studies that have specifically assessed the follow-up outcomes regarding opioid consumption, need for additional injections and/or pursuit of surgery are still scarce [21-23]. In a study aimed to compare clinical efficacy between interlaminar and transforaminal epidural injection in patients with axial pain due to cervical disc herniation, Lee *et al*. found favorable results in 2 weeks and moderate results in 8 weeks in patients with axial pain due to cervical disc herniation [24]. In a randomized, double-blind, active-controlled trial in order to assess the effectiveness of cervical interlaminar epidural injections of local anesthetic with or without steroids for the management of axial or discogenic pain, significant pain relief and functional improvement (≥50%) was present at the end of 2 years in 73% of patients receiving local anesthetic only and 70% receiving local anesthetic with steroids. Authors concluded that cervical interlaminar epidural injections with or without steroids might provide a significant improvement in pain and functioning in patients with chronic discogenic or axial pain that is function-limiting and not related to facet joint pain [25]. Another study by Benditz *et al*. aimed at showing the positive short-term effects of an inpatient multimodal pain management concept with the focus on cervical translaminar epidural steroid injection for patients with cervical radiculopathy. Fifty-four patients who had undergone inpatient multimodal pain management for ten days were evaluated before and after 10-days treatment. Neck pain was reduced by 57.4% and arm pain by 62.5%. 2 days after the epidural steroid injection, the pain was reduced by 40.1% in the neck and by 43.4% in the arms. Authors concluded that this modality seems to be an effective short-term approach to treating cervical radiculopathy [26]. In a prospective review study, Bush *et al*. evaluated 68 consecutive subjects suffering from cervical radiculopathy. Subjects received a fluoroscopically guided transforaminal cervical epidural steroid injection. Sixty-two percent of the cases had relief with transforaminal cervical epidural steroid injection. At the mean follow-up of 39 months (range 4–112), 76% had complete relief of arm pain, and 24% had a mean pain score of 2. Prior to treatment, 75% had a weakness. At follow-up 73% no longer experienced weakness. Eighty-four percent did not feel their symptoms interfered with their capacity to work [27]. In the present study, only 1/3 of participants underwent additional injections, and 10.8% proceeded to surgery, indicating a reduction in the need for invasive surgical treatment, which is consistent with previously performed studies [28]. In terms of patient satisfaction, previously performed interventions have shown a rating of more than average satisfaction. For example in a study by Park *et al*. on 128 patients, the patient satisfaction at 12 months after the procedure was reported excellent in 57 patients (44.5%), good in 65 patients (50.8%), and poor in one patient (0.8%) [11]. Some studies have concluded that repetitive steroid injections may reduce symptoms of pain and neck disability in patients with cervical radiculopathy at a short time follow-up [29, 30], however findings from our study state that even single injection of steroid in cervical epidural space can lead to significant pain reduction and neck disability improvement. As mentioned above, previous studies have reported pain reduction and improved activity after the cervical epidural steroid injection, both in short and long terms, which are consistent with findings from our study. However, the data on opioid consumption after cervical epidural steroid injection is less clear. In a study by Zarghooni *et al*., pain medication was significantly reduced after single-shot epidural steroid injection for radicular pain [31]. Initial studies by Rowlingson *et al*. regarding the conservative management of patients with cervical radiculopathy after epidural steroid injection showed a good or excellent response to cervical epidural steroid injection [32-34]. In a retrospective analysis of 20 cases of cervical radicular pain by Rathmal *et al*., outcomes of transforaminal epidural steroid injection were measured. The investigators reported pain reduction, and elimination in analgesic use in 60% of patients at 12–45 months’ follow-up [35]. To evaluate the long-term effectiveness of a single cervical epidural steroid injection performed with or without morphine, 24 patients suffering from chronic cervical radicular pain, were included in a prospective and randomized study. Results suggested that a single cervical epidural steroid injection performed in such cases produces long-lasting pain relief which is not improved when morphine is combined with steroid [36]. Another pilot study prospectively followed low back pain patients for three months after lumbar epidural steroid injection and estimated pain relief, function and opioid use over three months. Findings from their study revealed pain rating improvement and opioid decrease initially after lumbar epidural steroid injection for low back pain, but this effect was tapered over time [37]. However, in another study by Fridley *et al*., researchers show that opioid use did not decrease in the six months after epidural steroid injections [38]. In our study, the majority of patients were not using opioid after 2 years post-intervention, but since in this population, the use of opioids after epidural steroid injection is expected, and the fact that patients who received multiple injections were more likely to start taking opioids and to undergo lumbar surgery within the 6 months after treatment with epidural steroid injection. These findings can be concerning in terms of predicting future injections and/or spinal surgeries. The present study was one of the first researches evaluating patient-reported outcomes and satisfaction of cervical epidural steroid injections while comparing the effectiveness of between those who had undergone surgery and those who had not which serves as a strength of our works. However, due to the lack of equality of surgical and non-surgical patients, correlation of outcomes between these two categories cannot be calculated. Besides, the study was performed in a single pain intervention department and confirming the results by a relatively small number of patients examined in our study will require further prospective multi-central randomized trials with larger and clustered samples for accomplishing significant, clinically applicable results in interventional pain management settings.

## Conclusion

We demonstrate that cervical epidural steroid injection for cervical radiculopathy is an effective non-surgical treatment option, providing significant pain relief and functional improvement during 2-years follow-up along with higher-than-average patient satisfaction in most of our patients, to the extent that nearly 1/3 of patients needed additional injections and only 1/10 of the patients finally proceeded to surgery.

## Acknowledgment

We would like to thank Miss. Atefe Alaee for performing a comprehensive language edition that greatly improved the present manuscript.

## Conflict of Interest

No commercial party having a direct financial interest in the results of the research supporting this article has or will confer a benefit on the authors or on any organization with which the authors are associated.

**Table 1:** Participants’ Baseline and Demographic Characteristics.

**Variables**	**Frequency**	**Percentage**
**Sex**		
Male	12	32.4
Female	25	67.6
**No Comorbidity**	20	54.1
**Disc level**		
C4-5	5	13.5
C5-6	5	13.5
C6-7	3	8.1
C3-4, C4-5	1	2.7
C4-5, C5-6	4	10.8
C3-4, C4-5, C5-6, C6-7	1	2.7
C5-6, C6-7	13	35.1
C4-5, C5-6, C6-7	3	8.1
C3-4, C4-5, C5-6	2	5.4
**Protrusion site**		
Central	14	37.8
Left paracentral	8	21.6
Right paracentral	7	18.9
Central and left paracentral	7	18.9
Central and right paracentral	1	2.7
**Radiculation**		
Both upper extremity	13	35.1
Left upper extremity	14	37.8
Right upper extremity	9	24.3
Foraminal stenosis	20	54.1
Paresthesia	28	75.7
Positive Spurling test	31	83.8
Axial loading	20	54.1
Lhermitte’s Sign	10	27.0

**Table 2:** Frequency of Opioid Consumption, Additional Intervention, and Surgery

	Frequency (n=34)	Percentage
Opioid consumption	7	20.6%
Additional cervical epidural steroid injections	11	32.4%
Surgery	4	11.8%

**Table 3:** Comparison of Outcomes Stratified by Pursuit of Surgery

Variables		Progression to surgery (n=4)	No surgery (n=30)
NRS	Baseline	8.25	7.7
	After 2 years	7.5	4.6
NDI (at baseline)	Baseline	16.5	21.13
	After 2 years	26	16.23
PSQ (after 2 years)		1.5	3.4
Opioid consumption (during 2 years)		75%	13%
Additional injection (during 2 years)		75%	26%
